# The expression analysis of IL-6, IL-18, IL-21, IL-23, and TGF-β mRNA in the nasal mucosa of patients with Allergic rhinitis

**DOI:** 10.4314/ahs.v22i1.73

**Published:** 2022-03

**Authors:** Yousef Mirzaei, Zohreh Savari, Farshad Yazdani-Nafchi, Najmeh Salehi-Vanani, Elnaz Fallahi, Ashkan Pirayesh, Mohammadali Zahmati, Maryam Anjomshoa, Nader Bageri, Milad Sabzevary-Ghahfarokhi, Hedayatollah Shirzad, Mohamad Ali Zamani

**Affiliations:** 1 Department of Biogeosciences, Scientific Research Center, Soran University, Soran, Kurdistan Region, Iraq; 2 Department of Biology, Faculty of Basic Science, Islamic Azad University, Shahrekord Branch; 3 Cellular and Molecular Research Center, Basic Health Sciences Institute, Shahrekord University of Medical Sciences, Shahrekord, Iran; 4 The Clinical Biochemistry Research Center, Basic Health Science Institute, Shahrekord University of Medical Sciences, Shahrekord, Iran; 5 Department of Biology, North Tehran Branch, Islamic Azad University, Tehran, Iran; 6 Faculty of Veterinary Medicine, Tabriz Branch, Islamic Azad University, Tabriz, Iran; 7 Department of Otorhinolaryngology, Shahrekord University of Medical Sciences, Shahrekord, Iran

**Keywords:** Allergic rhinitis, inflammation, interleukin-23, Nasal Mucosa

## Abstract

**Background:**

The profile of inflammatory and suppressing cytokines is important to contribute to the disruption of TH1/TH2 balance in Allergic rhinitis (AR).

**Objective:**

This study aimed to assess the expression levels of IL-6, IL-18, IL-21, IL-23, and TGF-β in nasal biopsies in AR patients and evaluate its correlation with the severity of AR.

**Material and method:**

The study included 30 patients with mild persistent allergic rhinitis (MPAR), patients with moderate-to-severe (M/S) PAR, and 30 healthy individuals. The biopsies of nasal inferior turbinate mucosa were collected from each participant. The expression of IL-6, IL-18, IL-21, IL-23, and TGF-β was evaluated by the quantitative real-time polymerase chain reaction. The degree of eosinophil infiltration into the nasal mucosa, blood eosinophils, and total serum IgE level were also measured.

**Result:**

The expression of IL-6, IL-18, and IL-23 in patients with AR significantly increased compared to the control group. Conversely, the gene expression of the TGF-β declined in the M/S PAR group rather than the AR- group. The data did not show a significant difference in the expression of the IL-21 gene between AR+ and AR- groups.

**Conclusion:**

We suggested that inflammatory cytokines including IL-6, IL-18, and IL-23 may be involved in the severity of AR and associated with markers of inflammation.

## Introduction

Allergic rhinitis (AR) is a nasal mucosa inflammatory disorder characterized by an intense runny nose, rhinorrhea, mucus hypersecretion, and airway remodeling^1^. The prevalence of AR is ∼20% to 30% in adults and up to ∼40% in children.^2^. Uncontrolled AR is associated with insomnia or sleep disorders that affect the quality of life^3^. The diagnosis of AR is performed through the positive skin prick test (SPT) and/or serum specific IgE. The most common sensitizing aeroallergen in southwest Iran is weed pollen, molds, and animal dander^4^,^5^. The exposure to the allergen is mainly accompanied by unbalanced T helper 1 (Th1)/Th2 responses leading to releasing type-2 cytokines, such as interleukin (IL)-4, IL-5, and IL-13. These responses also recruit several immune cells to touch upon mast cells, eosinophils, macrophages, and neutrophils into the inflamed nasal mucosa^6^.

Interleukin (IL)-6 is one of the pre-inflammatory cytokines that can inhibit Th1 cell differentiation and induce the Th2 cells, particularly in allergic reactions^7^,^8^. Interestingly, it has been reported that epithelial cells isolated from asthma patients produce more IL-6 than healthy individuals^9^,^10^. Th1 cells that were treated by IL-18 were able to release more IFN-γ after exposure to allergens—e.g. Ovalbumin^11^. A study using atopic dermatitis animal models showed that high levels of IL-18 were released from skin-infiltrated mast cells and resulted in increased inflammation in the skin tissues^12^.

Transforming growth factor (TGF)-β1 is an inhibitory cytokine that can inhibit allergic reactions by stopping the IgE production and proliferation of mast cells 13. Furthermore, TGF-β1 plays an essential role as subepithelial fibrosis in chronic obstructive airway diseases^14^. IL 21—a pleiotropic α helical cytokine—is potentially produced by cells that are stimulated by TGF-β and IL 6 15,16. Through the inflammatory responses, this cytokine can develop autoimmune diseases; for instance, various studies have shown that by the time the mast cells were stimulated with IL 21, the release of histamine and other inflammatory mediators increased in vitro^17^. IL 21 is predominantly expressed by Th17 which has a high proportion in the peripheral blood of patients with AR. By producing IL-17, Th17 cells play a pivotal role in promoting eosinophil in the sensitization phase of mouse asthma^18^,^19^. IL-23 is another pro-inflammatory infiltrating neutrophil in the asthmatic mice airway, which its aberrant expression is associated with severe asthma20. Using an animal respiratory model, it has been shown that inhaled allergens elevated the expression of IL-23, leading to increased cytokines dependent on Th2. Conversely, when the IL-23 protein was neutralized, the severity of respiratory allergy symptoms decreased^21^.

In the previous study, we demonstrated that the expression of Fas—as a crucial factor for immune tolerance—decreased in AR patients in mRNA and protein levels^22^. For further investigation, the present study aimed to compare the mRNA levels of IL-6, IL-18, IL-21, IL-23, and TGF-β in nasal biopsies in the AR patients and the control individuals. Besides, the possible correlation between these cytokines and the severity of AR was evaluated.

## Material and method

### Patients and clinical specimens

This study was approved by the ethics committee of Shahrekord University of Medical Sciences, Shahrekord, Iran under the ethical code of “IR.SKUMS.REC.92-8-17.” All participants signed a written consent form. The study was assigned to 30 patients with AR and 30 healthy subjects. The patient group was divided into moderate/severe persistent AR (M/S PAR), mild persistent AR (M PAR) (Table 1). The Skin Prick Test in addition to the common examinations for medical symptoms (i.e. sneezing, rhinorrhea, and nasal congestion for at least 2 years) was used to confirm the diagnosis of AR^23^. The patients who had recurrent infections of the respiratory tract, the positive nasal culture of an infectious pathogen, positive history of smoking and consumption of systemic steroids or anti-allergic drugs (e.g. anti-histamine one week before nasal sampling) were excluded.

**Table 1:** Demographic information of the patients

Variables	Healthy control	mild persistent allergic rhinitis patients	moderate/severe persistent allergic rhinitis patients
Gender (male/female)	13/17	10/8	6/6
Age	25.8 ± (6.42)	26.7 ± (4.8)	26.0 ± (4.0)
Total IgE (IU/mL)	82.6 ± (43.8)	1061.1 ± (88.4)	236.3 ± (33.2)
Blood eosinophil count (absolute count)	74.5 ± (40.0)	618.9 ± (114.1)	255.7 ± (70.9)
The degree of eosinophil infiltration into the nasal mucosa (eos/HPF)	0.5 ± (0.5)	3.6 ± (1.8)	1.7 ± (0.59)
Disease duration (years)	-	17.1 ± (4.3)	15.1 ± (2.6)

Tissue specimens were obtained from the inferior turbinate mucosal of patients. Serum and complete blood count (CBC) were analyzed for the total and specific IgE measurement and Blood eosinophil counts, respectively. The degree of eosinophil infiltration into the nasal mucosa was assessed by hematoxylin and eosin (H&E) staining on the biopsy specimens^23^.

### RNA isolation and quantitative real-time PCR

Total RNA was extracted from the nasal tissue samples using Biozol solution (Bioflux; Japan). At 260/280 and 260/230 wavelength absorption ratios, the concentration of RNA was calculated by Nanodrop™ 2000c (Thermo Fisher Scientific, IL, USA). To cut down on any DNA contamination, 10 u/ml DNaseI were added to 1 mg/ml RNA samples for 30 min. Then, the Revert Aid firststrand cDNA synthesis kit (Thermo Scientific; Lithuania) was used to synthesize cDNA from total RNA by incubation at 25°C for 5 min, at 42°C for 1 h, and for 5 min at 72°C in a thermocycler. SYBR Green master mix (Takara; Japan) was used for the real-time PCR, which was performed in 35 cycles at a Rotor-Gene RG-300 (Corbett Research, Sydney, AU). Briefly, denaturation was carried out at 95°C for 10 min followed by incubation at 95°C for 15 s, 57°C for 20 s, and 72°C for 25 s. The specific primers listed in Table 2 were used to evaluate the expression of the TGF-β1, IL-6, IL-18, IL-21, and IL-23. β-actin gene was used to normalize the data. The standard curves were drawn to evaluate the primers' efficiency, which was above 90% on average. The data were expressed as fold change using the comparative cycle threshold (2-ΔΔct method).

**Table 2:** Demographic information of the patients

Variables	Healthy control	mild persistent allergic rhinitis patients	moderate/severe persistent allergic rhinitis patients
Gender (male/female)	13/17	10/8	6/6
Age	25.8 ± (6.42)	26.7 ± (4.8)	26.0 ± (4.0)
Total IgE (IU/mL)	82.6 ± (43.8)	1061.1 ± (88.4)	236.3 ± (33.2)
Blood eosinophil count (absolute count)	74.5 ± (40.0)	618.9 ± (114.1)	255.7 ± (70.9)
The degree of eosinophil infiltration into the nasal mucosa (eos/HPF)	0.5 ± (0.5)	3.6 ± (1.8)	1.7 ± (0.59)
Disease duration (years)	-	17.1 ± (4.3)	15.1 ± (2.6)

*Results are shown as mean ± SD (standard deviation).

### Statistical analysis

All data were analyzed according to the paired t-test. All data are represented as mean ± standard deviation (SD) of three independent experiments. The P-value <0.05 was considered to indicate a statistically significant result. All statistical analyses were performed using SPSS version 24.0 (IBM Corp., Armonk, NY, USA) and GraphPad Prism 8.0 (GraphPad Software, Inc., La Jolla, CA, USA).

## Result

### Clinical characteristics

The demographic information of AR+ (AR patients) and AR- (healthy control group) are indicated in Table 1. The age of patients was matched with healthy control individuals. The eosinophil counts in the CBC test and the degree of eosinophil infiltration into the nasal mucosa were higher than the control group. Also, serum total IgE level was lower in the control group—at about 82.6 ± 43.8 IU/mL—than the M/S PAR group which was determined around 236.3 ± 33.2 IU/mL (Table 1).

### The Gene Expression of IL-6, IL-18, and IL-23 increased in Nasal Mucosa

**Fig. 1:**
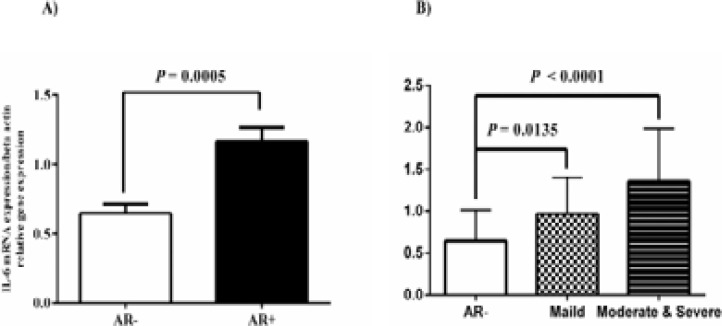
IL-6 mRNA level was evaluated in the nasal mucosa of 30 paired AR and healthy individuals. A) IL-6 gene expression in AR- and AR+ was shown. The data from the real-time PCR for human IL-6 were normalized versus β-actin. B) IL-6 gene was significantly overexpressed in biopsies of M/S PAR patients than AR+ group. P-values less than 0.05 were statistically significant. In this figure: AR: Allergic Rhinitis; M/S PAR: moderate/severe persistent allergic rhinitis; M PAR: mild persistent allergic rhinitis. We set the level of statistical significance to P-value <0.05.

**Fig. 2:**
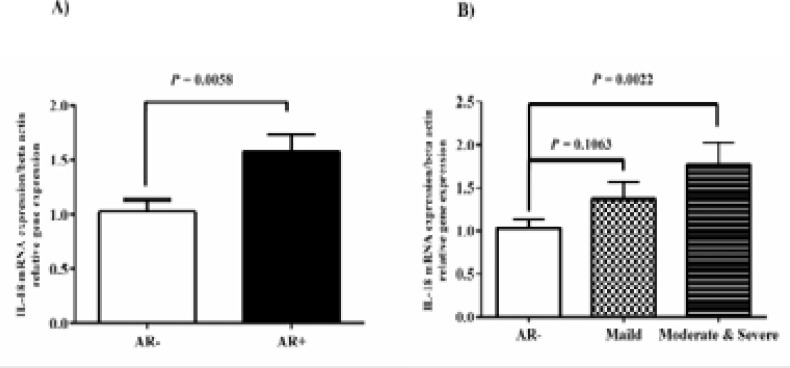
IL-18 mRNA level was evaluated in the nasal mucosa of 30 paired AR and healthy individuals. A) IL-18 gene expression in AR- and AR+ was shown. B) IL-18 gene significantly increased in biopsies of M/S PAR patients rather than the AR- group. We set the level of statistical significance to P-value <0.05.

We also evaluated the mRNA level of IL-6, IL-18, IL-21, and IL-23 by qRT-PCR in the nasal mucosa of AR patients and healthy individuals. In this experiment, the gene expression of IL-6 in the AR+ group was twofold higher than in the AR- group (P-value= 0.005; Fig. 1A). The differential expression of IL-6 indicated that the gene expression in M/S PAR patients seemed to be more than M PAR patients samples (Fig. 1B). The IL-18 mRNA level was remarkably elevated in the AR+ group compared to the AR- group (Fig 2). Interestingly, the gene expression of IL-18 increased in M/S PAR and M PAR than in the AR- group (P-value = 0.0163 and 0.0022; Fig. 2B).

**Fig. 3:**
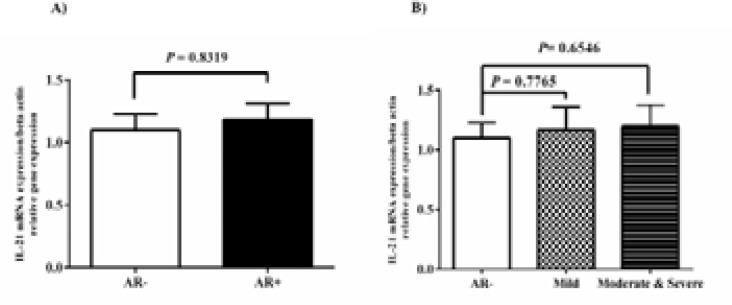
IL-21 mRNA level was evaluated in the nasal mucosa of 30 AR and 30 healthy individuals. A) We did not detect any significant difference in mRNA level of IL-21 between AR- and AR+ groups. B) The comparison of IL-21 gene expression between M PAR & M/S PAR groups and AR- group did not show any statistical signification (P-value > 0.05). We set the level of statistical significance to P-value <0.05

**Fig. 4:**
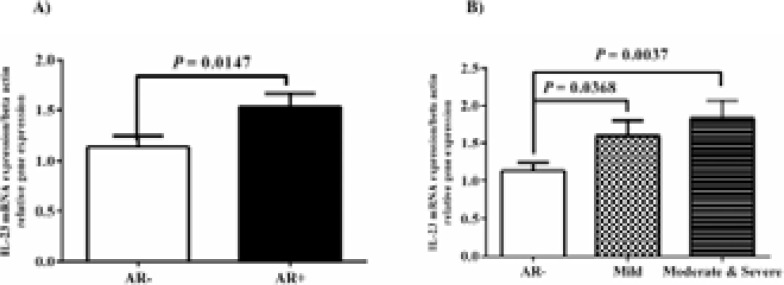
IL-23 mRNA level was evaluated in the nasal mucosa of 30 AR and 30 healthy control. A) The gene expressions of IL-23 were compared between AR- and AR+ groups. B) IL-23 mRNA level was evaluated in nasal mucosa biopsies of M/S PAR group than AR- group. We set the level of statistical significance to P-value <0.05.

The comparison of gene expression of the IL-21 between AR- and AR+ nasal mucosa did not show any statistically significant data (P-value > 0.05). Likewise, no difference in expression of IL-21 gene between M/S PAR groups and AR- group (P-value >0.05; Fig. 3) was observed. The IL-23 mRNA level increased in the AR+ group compared to the AR- group (P-value = 0.0147; Fig. 4A). The statistical analysis revealed the meaningful differences between M/S PAR and AR- groups (P-value= 0.0037).

### The Expression of TGF-Β1 decreased in Nasal Mucosa of AR Patients

**Fig. 5:**
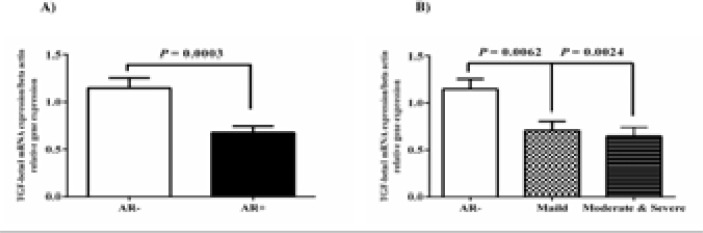
TGF-β mRNA level was evaluated in the nasal mucosa of 30 AR and 30 healthy individuals. A) The comparison of TGF-β1 gene expressions between AR- and AR+ groups. B) TGF-β mRNA level decreased in nasal mucosa biopsies of M/S PAR group than AR- group. We set the level of statistical significance to P-value <0.05.

Based on the results of qRT-PCR, TGF-β gene expression decreased in the nasal mucosa of patients with AR (AR+) compared to the healthy control group (AR-) (P-value = 0.0003; Fig. 5A). Surprisingly, Fig. 5B showed that the mRNA level of TGF-β in the M/S PAR group was statistically lower than AR- group (P-value= 0.0024); however, this level was slightly equal in both M PAR and M/S PAR groups.

## Discussion

AR is characterized by a disturbance in nasal mucosal immune tolerance and a deviation in the immune response to Th2 reactions^24^. To determine whether the activation of these cells causes the secretion of inflammatory cytokines or not, we analyzed the profile of the inflammatory cytokines. Moreover, the relation between inflammatory cytokine and the occurrence of manifestations pertinent to the severity was evaluated in M/S PAR and M PAR groups. In our study, we found higher levels of IL-6 in the nasal mucosa of AR patients. IL-6 in nasal mucosa was higher in M/S PAR in comparison with the M PAR. Conflicting findings have been obtained in allergic animal trials. In mice where the IL-6 gene was knocked out, the progressive inflammatory airway was observed, while the low degree of inflammation in the airways was seen in animal models using inhibitory antibodies against IL-6 25. There have been several reports of elevated IL-6 protein in serum and biopsy specimens in people with asthma compared with the control group^26^,^27^. Increased expression of this protein in the epithelial cells of the nasal mucosa in patients was documented in a study of patients with rhinitis^28^. In addition, IL-6 elevated the survival and expansion of Th-17 cells in the allergic airway responses. It is suggested that the engagement of soluble IL-6 receptors contribute to a trans-signaling pathway that in turn support Th2 and Th-17 cell differentiation^29^. A high level of IL-17 expression in patients with allergic and viral rhinitis may lead to high infiltrated eosinophils and neutrophils in inflammatory lesions^19^.

Some scrapes of evidence have indicated a positive association between local and/or circulating IL-18 levels and various types of allergy such as AR, asthma, and atopic dermatitis^30^,^31^. In the present study, IL-18 levels were dramatically high in M/S PAR group, reflecting that this cytokinemight be involved in the pathogenesis of allergic asthma. Research on asthma rat models found that IL-18 injection substantially exacerbated the symptoms of asthma^32^. Another study reported that the severity of allergic reactions can be decreased if the IL-18 signaling was blocked^33^. The secretion of IL-18 in nasal secretions increases, particularly in cases of asthma after stimulation with domestic allergens and outdoor allergens^34^. Smith et al. have proved the role of IL-18 in stimulating the production of Th2 cytokines such as IL-13, IL-9, and IL-4. IL-18 can function as an endogenous factor, which has a direct role in the incidence of allergic and autoimmune diseases^35^. It has also been indicated that the function of IL-18 enhances inflammatory factors and suppresses the IL-10 regulatory factor in vitro^36^.

In contrast to inflammatory cytokines, a decreased secretion of TGF-β1 was shown in patients with AR+, particularly in the M/S PAR group. Low levels of TGF-β1 as an inhibitory cytokine are more likely to result in the enhanced function of Th1, Th2, and Th17 cells. Slib et al. found that a decrease in protein expression was measured in people with seasonal and permanent AR compared with non-allergic individuals^37^. Studies by Nakanishi et al. found a link between TGF-β protein expression and the incidence of asthma^38^; however, this relation has not been confirmed in a study carried out by Hoshin et al^39^. TGF-β and IL-6 Activate the development of IL-21 simultaneously. Although the expression of IL-21 increased in AR+ patients in our experiment, this increase was not statistically significant. Regarding the role of IL-21 in allergies, there have been conflicting effects on eosinophil cells. Suto et al. reported that the injection of IL-21 protein into allergy animal models decreased the number of eosinophilic cells—which are essential allergy-related cells—at the site of inflammation by reducing specific IgE levels. Similar results were also observed from injections of IL-21 into the anaphylactic mice and AR models^40^,^41^. Conversely, Frohlich et al. have seen a decrease in eosinophilia in the allergic animal model that their IL-21 receptor was knocked down^42^.

The existence of pro-inflammatory mediators expressed in the airway inflammation illustrates the role of specific cytokine expressed in the epithelial airway. The pro-inflammatory cytokine IL-23 potentially contributes to autoimmune diseases via integrating innate and adaptive immune systems into the airway mucosal layer^43^,^44^. The biopsies of nasal mucosa in the AR+ group showed a significant increase than AR- group. Beyond that, the statistical analysis showed meaningful differences between the M/S PAR group and AR- group. IL-23 and its receptor potentially contribute to Th2 differentiation by regulating airway inflammation in an independent manner to IL-17^45^. In a study of animal models of asthma, Peng et al. demonstrated that this protein plays a crucial role in the development of asthma. In this study, mice with a defective il-23 gene showed lower allergic airway inflammation, while transgenic overexpression of IL-23R increased the severity of allergy symptoms^46^. Other studies in mouse models of asthma have emphasized upon the role of IL-23 in the development of allergies (30). In addition, eosinophilia in old male FVB mice could be prevented if IL-23 was suppressed^29^.

## Conclusion

The possible role of IL-6, IL-18, and IL-23 in the AR severity was indicated in this study. These mediators may be used as biomarkers for the severity of inflammation in PAR. Besides, the role of suppressing cytokines and TGF-β, in particular, can help to shed light on the pathogenesis of AR. However, the precise molecular mechanism taking part in the allergic airway remains to be explained. We believe that future studies could fruitfully explore such mechanisms to help the AR patients.

## Declarations

### Abbreviations

AR: Allergic rhinitis; M PAR: Mild persistent allergic rhinitis; M/S PAR: Moderate to Severe persistent allergic rhinitis; SPT: skin prick test; Th1: T helper1; IL-4: Interleukin-4; TGF-β1: Transforming growth factor-1; TNSS: Total Nasal Syndrome Score; CBC: complete blood count.
